# Supplementary material for: Factors influencing users’ willingness to use new energy vehicles
Source: PLoS One. 2023 May 18;18(5):e0285815. doi: 10.1371/journal.pone.0285815 (PMC10194972; doi:10.1371/journal.pone.0285815)
Supplement: S2 File — (DOCX) [file pone.0285815.s002.docx]

**Factors influencing users’ willingness to use new energy vehicles**

Notes: new energy vehicles (NEV)

1. **Your gender？**

- Male
- Female

1. **Your age？**

- Under 30
- 31-40
- 41-50
- Above50

1. **Your education background？**

- High school graduates or under
- Vocational school graduates
- Undergraduates
- Master students or above

1. **Your profession？**

- Company Employees
- Students
- Public institutions
- Self-employed
- Civil servants
- Others

1. **I think driving an NEV is more efficient and relaxing？**

- Definitely agree
- Agree
- Neither
- Disagree
- Definitely disagree

1. **I think an NEV offers better driving experience and is more comfortable？**

- Definitely agree
- Agree
- Neither
- Disagree
- Definitely disagree

1. **I think an NEV makes communication more convenient and smoother？**

- Definitely agree
- Agree
- Neither
- Disagree
- Definitely disagree

1. **I think an NEV makes car life richer and exciting？**

- Definitely agree
- Agree
- Neither
- Disagree
- Definitely disagree

1. **I think an NEV with self-learning ability knows me better and meets my personalized needs？**

- Definitely agree
- Agree
- Neither
- Disagree
- Definitely disagree

1. **I think it’s easier to drive an NEV because it does not require frequent checking of the instructions？**

- Definitely agree
- Agree
- Neither
- Disagree
- Definitely disagree

1. **I think the operation in an NEV is more convenient？**

- Definitely agree
- Agree
- Neither
- Disagree
- Definitely disagree

1. **I think the operation interface in an NEV is friendly and easy to identify？**

- Definitely agree
- Agree
- Neither
- Disagree
- Definitely disagree

1. **I think all the intelligent functions in an NEV are user-friendly and useful？**

- Definitely agree
- Agree
- Neither
- Disagree
- Definitely disagree

1. **I think it’s very convenient to do remote software update for an NEV？**

- Definitely agree
- Agree
- Neither
- Disagree
- Definitely disagree

1. **I think an NEV is prone to spontaneous combustion？**

- Definitely agree
- Agree
- Neither
- Disagree
- Definitely disagree

1. **I think it’s easy for an NEV to leak privacy？**

- Definitely agree
- Agree
- Neither
- Disagree
- Definitely disagree

1. **I think the autonomous driving of an NEV of is prone to error？**

- Definitely agree
- Agree
- Neither
- Disagree
- Definitely disagree

1. **I think it’s easy to decrypt the digital key system of an NEV？**

- Definitely agree
- Agree
- Neither
- Disagree
- Definitely disagree

1. **I think an NEV is easy for hackers to attack and therefore not safe？**

- Definitely agree
- Agree
- Neither
- Disagree
- Definitely disagree

1. **I think the overall cost of purchasing and using an NEV is high？**

- Definitely agree
- Agree
- Neither
- Disagree
- Definitely disagree

1. **I think charging piles are scarce and the charging is costly and time consuming？**

- Definitely agree
- Agree
- Neither
- Disagree
- Definitely disagree

1. **I think it’s costly to fix an NEV？**

- Definitely agree
- Agree
- Neither
- Disagree
- Definitely disagree

1. **I believe that the batteries of NEVs wear out easily, the replacement cost is high, and the car retention rate is low？**

- Definitely agree
- Agree
- Neither
- Disagree
- Definitely disagree

1. **I think the insurance premium for an NEV are much higher than for gasoline cars？**

- Definitely agree
- Agree
- Neither
- Disagree
- Definitely disagree

1. **I think NEVs offer many amusing features like games, videos and music？**

- Definitely agree
- Agree
- Neither
- Disagree
- Definitely disagree

1. **I think NEVs have many novel and exciting intelligent features？**

- Definitely agree
- Agree
- Neither
- Disagree
- Definitely disagree

1. **I think NEVs are interesting for multiple human-machine interactions？**

- Definitely agree
- Agree
- Neither
- Disagree
- Definitely disagree

1. **I think NEVs have many offline groups and events, which is attractive to me？**

- Definitely agree
- Agree
- Neither
- Disagree
- Definitely disagree

1. **I think NEVs provide young, fashionable and cool outlook, which is quite unique？**

- Definitely agree
- Agree
- Neither
- Disagree
- Definitely disagree

1. **Media reports on low carbon and environment protection affect my BI to accept NEVs？**

- Definitely agree
- Agree
- Neither
- Disagree
- Definitely disagree

1. **Media reports on young culture affect my BI to accept NEVs？**

- Definitely agree
- Agree
- Neither
- Disagree
- Definitely disagree

1. **Media Reports on smart technology will affect my BI to accept NEVs？**

- Definitely agree
- Agree
- Neither
- Disagree
- Definitely disagree

1. **Media Reports on safety and reliance will affect my BI to accept NEVs？**

- Definitely agree
- Agree
- Neither
- Disagree
- Definitely disagree

1. **Media Reports on customer-centered will affect my BI to accept NEVs？**

- Definitely agree
- Agree
- Neither
- Disagree
- Definitely disagree

1. **People around me think it’s cheap to fuel up an NEV, which is quite suitable for me ,as I drive a lot？**

- Definitely agree
- Agree
- Neither
- Disagree
- Definitely disagree

1. **People around me think an NEV offers superb driving experience, which is quite suitable for me？**

- Definitely agree
- Agree
- Neither
- Disagree
- Definitely disagree

1. **People around me think NEVs are smart and convenient, which is quite suitable for me？**

- Definitely agree
- Agree
- Neither
- Disagree
- Definitely disagree

1. **People around me think NEVs are environment-friendly and emit less carbon, which is quite suitable for me？**

- Definitely agree
- Agree
- Neither
- Disagree
- Definitely disagree

1. **People around me think NEVs are safe and reliable , so they encourage me to buy？**

- Definitely agree
- Agree
- Neither
- Disagree
- Definitely disagree

1. **I probably will drive an NEV in the future？**

- Definitely agree
- Agree
- Neither
- Disagree
- Definitely disagree

1. **I am driving and will continue to drive an NEV？**

- Definitely agree
- Agree
- Neither
- Disagree
- Definitely disagree

1. **I recommend others drive an NEV？**

- Definitely agree
- Agree
- Neither
- Disagree
- Definitely disagree

1. **I will switch to an NEV if they are safer and more reliable？**

- Definitely agree
- Agree
- Neither
- Disagree
- Definitely disagree

1. **I will switch to an NEV if they are smarter and technology-intensive？**

- Definitely agree
- Agree
- Neither
- Disagree
- Definitely disagree
